# Construction of an easy-to-use CRISPR-Cas9 system by patching a newly designed EXIT circuit

**DOI:** 10.1186/s13036-017-0072-5

**Published:** 2017-09-04

**Authors:** Qiang Tang, Chunbo Lou, Shuang-Jiang Liu

**Affiliations:** 1 State Key Laboratory of Microbial Resources and Environmental Microbiology Research Center, Chinese Academy of Sciences, Beijing, 100101 China; 2 University of Chinese Academy of Sciences, Beijing, 100049 China; 3 CAS Key Laboratory for Microbial Physiology and Metabolic Engineering, Chinese Academy of Sciences, Beijing, 100101 China; 4 Institute of Microbiology, Chinese Academy of Sciences, Beichen Xilu 1, Chaoyang District, Beijing, 100101 China

**Keywords:** EXIT circuit, I*-Sce*I, Plasmid elimination, One-step, CRISPR-Cas9, Easy-to-use, Genome editing, Atrazine-degradation, *Escherichia coli*

## Abstract

**Background:**

Plasmid-borne genetic editing tools, including the widely used CRISPR-Cas9 system, have greatly facilitated bacterial programming to obtain novel functionalities. However, the lack of effective post-editing plasmid elimination methods impedes follow-up genetic manipulation or application. Conventional strategies including exposure to physical and chemical treatments, or exploiting temperature-sensitive replication origins have several drawbacks (e.g., they are limited for efficiency and are time-consuming). Therefore, the demand is apparent for easy and rapid elimination of the tool plasmids from their bacterial hosts after genetic manipulation.

**Results:**

To bridge this gap, we designed a novel EXIT circuit with the homing endonuclease, which can be exploited for rapid and efficient elimination of various plasmids with diverse replication origins. As a proof of concept, we validated the EXIT circuit in *Escherichia coli* by harnessing homing endonuclease I-*Sce*I and its cleavage site. When integrated into multiple plasmids with different origins, the EXIT circuit allowed them to be eliminated from the host cells, simultaneously. By combining the widely used plasmid-borne CRISPR-Cas9 system and the EXIT circuit, we constructed an easy-to-use CRISPR-Cas9 system that eliminated the Cas9- and the single-guide RNA (sgRNA)-encoding plasmids in one-step. Within 3 days, we successfully constructed an atrazine-degrading *E. coli* strain, thus further demonstrating the advantage of this new CRISPR-Cas9 system for bacterial genome editing.

**Conclusions:**

Our novel EXIT circuit, which exploits the homing endonuclease I-*Sce*I, enables plasmid(s) with different replication origins to be eliminated from their host cells rapidly and efficiently. We also developed an easy-to-use CRISPR-Cas9 system with the EXIT circuit, and this new system can be widely applied to bacterial genome editing.

**Electronic supplementary material:**

The online version of this article (doi:10.1186/s13036-017-0072-5) contains supplementary material, which is available to authorized users.

## Background

Genome editing tools and editing strategies have enabled bacteria to be programed for different functionalities and their genetic architectures are engineered to make microbial cell factories for generating products for humans [1, 2], to provide workhorses for environmental clean-up [3, 4], or to build vehicles for vaccine delivery [5, 6]. The genetic tools developed on plasmid platforms include genome editing [7, 8], large fragment integration [9, 10], and experimental evolution [11, 12]. Although great conveniences are brought by plasmid-borne tools, eliminating such plasmids is an obstacle that impedes follow-up genetic manipulation or application. Physical methods such as microwaving [13] and chemical methods such as exposure to clorobiocin [14] were utilized to eliminate the tool plasmids. However, their practical uses are limited for low efficiency and potential damages to chromosomal DNA molecules. Another solution for plasmid removal is the use of counter-selection markers, but this approach often involves in genetic modification of host strain, and is not applicable for strains other than the modified host [15]. Utilizing temperature-sensitive origins of replication to build plasmids is a widely used strategy for genome editing, and examples of this include pW01ts for *Clostridium ljungdahlii* [16], pWV01 for *Lactococcus lactis* [17], and pSC101 for *Escherichia coli* [18]; however, this method of plasmid elimination has low efficiency and is time-consuming. Additionally, plasmids with temperature-sensitive replication origins often have low copy numbers [18, 19], a factor that often limits the expression of target proteins as well as the efficiency of the editing tools. Moreover, widely used two-plasmid systems [20, 21] require at least two different types of plasmid replication origins to avoid plasmid incompatibility [22].

The clustered regularly interspaced short palindromic repeats-(CRISPR)-associated system (CRISPR-Cas system) has emerged as an efficient genome editing technology in several prokaryotes and eukaryotes, including *E. coli* [10, 23], *Saccharomyces cerevisiae* [24], plants [25] and mammalian cells [26]. The type II CRISPR-Cas system from *Streptococcus pyogenes* uses a maturation CRISPR RNA (crRNA) and trans-activating crRNA (tracrRNA) or fused crRNA and tracrRNA as a single synthetic guide RNA (sgRNA) guiding the nuclease Cas protein 9 (Cas9) to the target of any DNA sequence, known as a protospacer, with a protospacer-adjacent motif (PAM) present at the 3′ end (NGG, where N represents any nucleotide) [27]. The 20-bp complementary region (N20) with the requisite NGG PAM that matches the genomic loci of interest is programmed directly into a heterologously expressed CRISPR array or synthetic guide RNA (sgRNA) transcript. Typically, the CRISPR-Cas9 system is programmed on two plasmids for bacterial genome editing: one encodes the Cas9 endonuclease, while the other encodes sgRNA to target a specific DNA sequence [8, 28–31]. However, eliminating the encoding plasmids can be an issue in terms of the time and screening required [28]. In *E. coli,* elimination of the CRISPR-Cas9 plasmids after genome editing has been attempted. Jiang et al. [30] and Li et al. [32] used inducible promoters to control the expression of the gRNA plasmid-targeting sgRNA, and assembled the structure to the Cas9 encoding plasmids. In order to eliminate the system, firstly the gRNA plasmid-targeting sgRNA is induced, leading Cas9 nuclease to cleave and eliminate the gRNA plasmid. Afterwards, the Cas9 plasmid, which was built with the temperature sensitive origin pSC101, is eliminated by changing the cultivation temperature. Ronda et al. designed a self-killing plasmid to carry the gRNAs [33]. An L-rhamnose inducible CRISPR natural array encoding two pre-crRNAs that target the origin and the kanamycin antibiotic marker of the plasmid. Upon induction, the gRNA plasmid was cut and digested to facilitate sequential engineering cycles. In another study, Reisch et al. [31] utilized the temperature sensitive pSC101 replication origin to encode sgRNA. To cure the system, the pSC101-encoded sgRNA targeting the Cas9 plasmid was transformed to eliminate the Cas9 encoding plasmid, and this plasmid was then cured via the temperature sensitive pSC101 replication origin. However, with both of the aforementioned strategies, the CRISPR-Cas9 encoding plasmids have to be eliminated sequentially, consuming at least two days.

In this study, we exploited the rare-cutting restriction feature of the homing endonuclease and its cognate recognition site [34, 35] to rationally design a synthetic circuit to eliminate one or more target plasmids simultaneously (designated the EXIT circuit). The yeast mitochondrial I-*Sce*I endonuclease, which has an 18-bp recognition site (5′-TAGGGATAACAGGGTAAT-3′), has been employed to introduce specific double-strand DNA breaks (DSBs) in many organisms [36–39]. As a proof of concept, we constructed the EXIT circuit in *E. coli* by harnessing the homing endonuclease I-*Sce*I and its recognition site in a modularized fashion. The modularized EXIT circuit can be rapidly assembled to different plasmids in a “plug-and-play” way. By patching this novel EXIT circuit, we reconstructed an easy-to-use CRISPR-Cas9 system that enabled both the Cas9- and the sgRNA-encoding plasmids to be eliminated in a single step. The new easy-to-use CRISPR-Cas9 system facilitated editing of the *E. coli* genome, and a recombinant *E. coli* strain capable of degrading the herbicide atrazine [40, 41] was rapidly constructed. Our results show that the EXIT circuit is a simple, reliable and rapid method for one-step plasmid elimination, and that the reconstructed CRISPR-Cas9 system is an easy-to-use and efficient editing tool for bacterial genomes.

## Results

### Designing and constructing the synthetic EXIT circuit

**Fig. 1:**
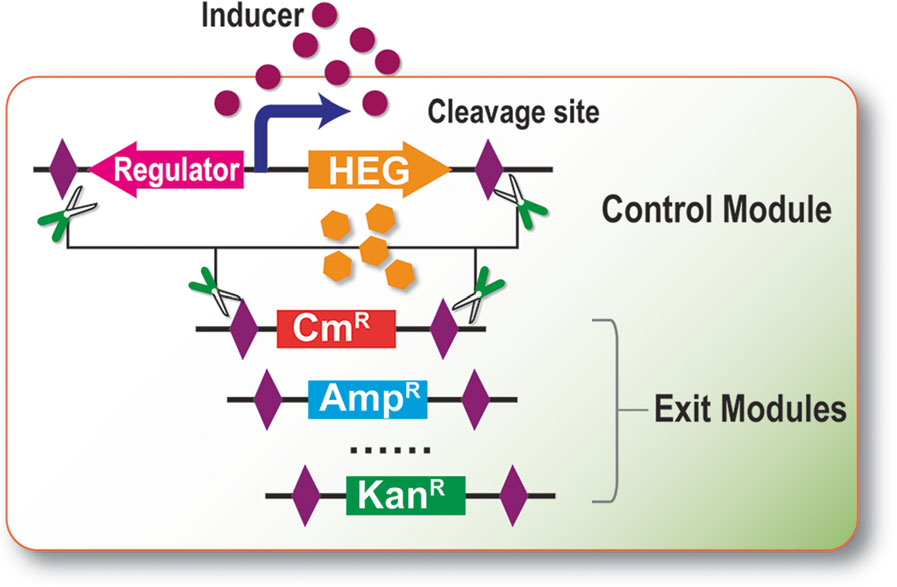
Conceptual illustration of the EXIT circuit. The EXIT circuit comprises a control module and an exit module. The two modules can be assembled into one or more plasmids. Activation of the homing endonuclease gene (HEG) will result in cleavage of the control and exit modules, thereby eliminating the host plasmids

To rapidly eliminate plasmids from their bacterial hosts, the homing endonuclease and its cognate recognition site [34, 35] were exploited to construct the synthetic EXIT circuit. As outlined in Fig. 1, the EXIT circuit comprises a control module and an exit module. The control module consists of the homing endonuclease under the control of a tightly-regulated promoter, with two terminators flanking both sides to insulate the cassette from adjacent sequences, and two cognate endonuclease recognition sites at both ends. The exit module has an antibiotic resistance cassette bordered by two homing endonuclease recognition sites. When the tightly regulated promoter is activated by the inducer, the homing endonuclease recognizes and cleaves its recognition sites in the control and exit modules, resulting in DSBs and plasmid elimination. By integrating the two modules, the plasmid(s) can be efficiently eliminated from the host. The EXIT circuit can be integrated into plasmids with any replication origin, thus allowing multiple plasmids present in the same cell to be simultaneously eliminated later on.

**Fig. 2:**
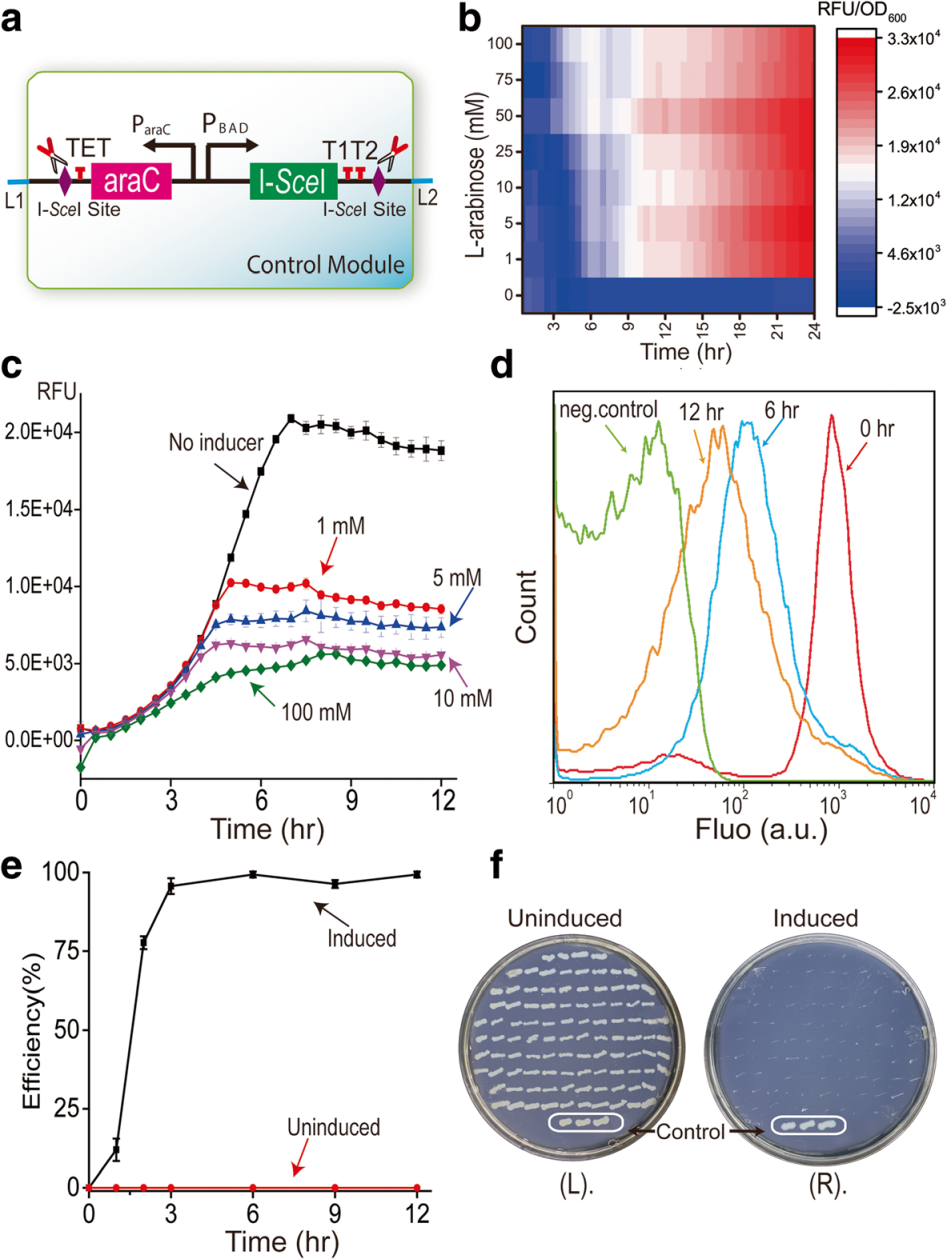
Validation of the control module of the EXT circuit. **a** Schematic of the control module of the EXIT circuit constructed in *E. coli*. **b** Characterization of I-*Sce*I expression from the I-*Sce*I-GFP*mut3b* pEC001 plasmid in response to different concentrations of the L-arabinose inducer. **c**-**d** Evaluation of control module responses to different L-arabinose concentrations at the population level (**c**) and at the single-cell level (**d**). **e** Elimination efficiencies of the control module at different induction times. **f** Visual demonstration of elimination efficiency on plates. Symbols in Panel **c**: (■) No inducer and (●) 1 mM; (▲) 5 mM; (▼) 10 mM; (♦) 100 mM L-arabinose. For panels **c** and **e**, three samples were evaluated and the standard errors are indicated

As a proof of concept, we employed the I-*Sce*I homing endonuclease and its cleavage site to build the EXIT circuit in *E. coli* (Fig. 2a). The pEC101 plasmid with the p15A-origin [42] that carries the EXIT circuit was created. In this EXIT circuit, the functional I-*Sce*I endonuclease was tightly controlled under an L-arabinose-inducible promoter P_BAD_ [43], with two terminators (TET and T1 T2) added to both sides and two I-*Sce*I cleavage sites inserted at both ends. Two linkers (L1 and L2) from R2oDNA Designer [44] were used for the easy assembly of plasmids with various replication origins in a plug-and-play fashion.

The expression level of the I-*Sce*I endonuclease (under control of the P_BAD_ promoter) was evaluated using the pEC001 I-*Sce*I-GFP*mut3b* fusion plasmid. As shown in Fig. 2b, I-*Sce*I expression increased with increasing concentrations of L-arabinose (0–100 mM). L-arabinose at 100 mM showed the fastest and greatest response for I-*Sce*I expression.

### Evaluating the EXIT circuit

To determine the plasmid elimination efficiency of the EXIT circuit, the GFP*mut3b* reporter gene was used as an indicator. GFP*mut3b* equipped with a strong constitutive promoter, was inserted into the pEC101 plasmid to generate plasmid pEC201. When the EXIT circuit was activated by L-arabinose, the I-*Sce*I endonuclease cleaved its cognate sites and the plasmid was killed. As a result, the fluorescence from GFP*mut3b* decreased. We evaluated the elimination efficiencies at both the population (Fig. 2c) and single-cell levels (Fig. 2d).

First, we characterized the elimination efficiency of the EXIT circuit at the population level using different concentrations of L-arabinose as the inducer. As shown in Fig. 2c, we found that 1 mM L-arabinose induced a dramatic decrease in GFP*mut3b* fluorescence. However, increasing the L-arabinose concentrations up to 100 mM resulted in the most significant and fastest decrease of fluorescence, suggesting that the plasmids were more efficiently and rapidly eliminated (Fig. 2c). Second, we tested the dynamic response of the EXIT circuit at the single-cell level (Fig. 2d). The average GFP*mut3b* fluorescence decreased significantly and continuously from 0 h, when L-arabinose was added, to 12 h afterwards. We also determined the average fluorescence of the cells at 18 h post-treatment (see Additional file 1: Figure S1) and the results showed that it was similar to that at 12 h. That some cells retained fluorescence might be caused by a remnant of the GFP*mut3b* protein remaining in the cells.

We further evaluated the elimination efficiency of the EXIT circuit by monitoring the loss of chloramphenicol resistance in recombinant *E. coli* harboring pEC101. It was expected that *E. coli* would fail to grow on chloramphenicol plates when pEC101 was eliminated. Samples were taken at 0, 1, 2, 3, 6, 9 and 12 h, and the results indicated that approximately 100% elimination was achieved at 3 h and afterwards (Fig. 2e). A representative determination of plasmid elimination in uninduced and induced cells (at 12 h) is shown in Fig. 2f. The EXIT circuit-encoding plasmid was very stable even without antibiotic selection pressure in the uninduced sample (Fig. 2f, panel L); however, it was rapidly and efficiently eliminated when the EXIT circuit was activated by L-arabinose (Fig. 2f, panel R). Elimination of the pEC101 plasmid was confirmed by PCR verification with the pEC101-specific primer pair (Additional file 1: Figure S2A).

### Validation of the EXIT circuit using different plasmid origins and simultaneous elimination of two plasmids

**Fig. 3:**
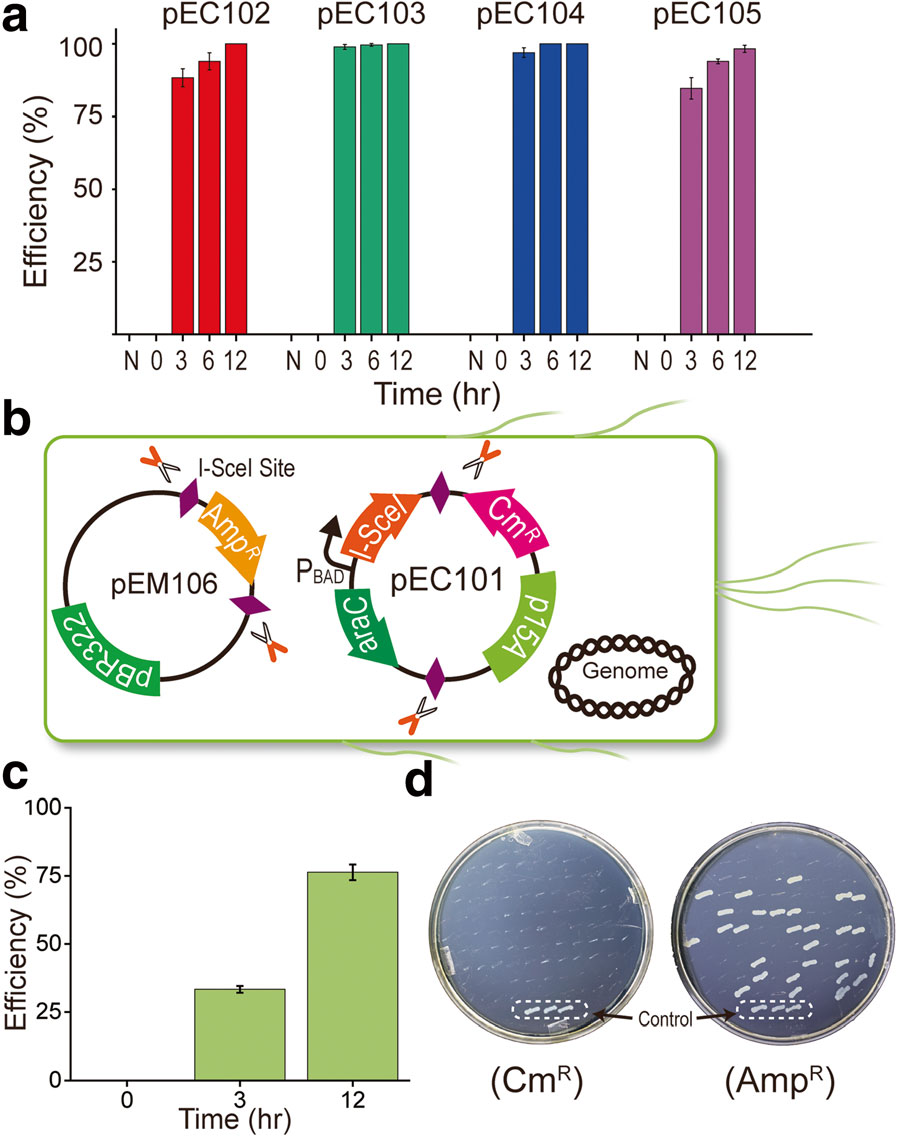
Validation of the EXIT circuit with different plasmid replication origins and simultaneous plasmid elimination. **a** Robustness of the EXIT circuit with four different plasmid replication origins. N: not induced. **b** Scheme used for one-step elimination of two plasmids. **c** Efficiency determination for eliminating both pEC101 and pEM106 (bar represents standard error; *n* = 3 independent cultures). **d** Visual demonstration of pEC101 and pEM106 elimination on plates

The modularity and orthogonality of the functional elements of the newly designed EXIT circuit means that this circuit may be readily applicable to a wide variety of plasmids with different replication origins. To confirm this, the EXIT circuit was assembled in other plasmid types with different replication origins, and four plasmids (pEC105, pEC102, pEC103 and pEC104) were constructed from pBelobac11 (single copy) [45], pBR322 (medium copy) [46], colE1 (high copy) [47] and pMB1 (very high copy) [48], respectively. The elimination efficiencies of these plasmids were determined (Fig. 3a), and the results indicated that all four plasmids were eliminated at high efficiency even after only a 3 h induction. Approximately 100% plasmid elimination was observed after a 12 h induction. The plasmid elimination was also confirmed by PCR (Additional file 1: Figure S2, B-E). The results indicate that the EXIT circuit possesses the important feature of robustness across various plasmids.

Another important feature of the EXIT circuit is its ability to allow multiple plasmids in the same cell to be eliminated simultaneously. To demonstrate this feature, the control module and the exit module of the EXIT circuit were assembled in two plasmids. The plasmid encoding the I-*Sce*I endonuclease (Control module, Fig. 1) was designated the elimination plasmid, while the other plasmid encoding the antibiotic cassette flanked by two I-*Sce*I cleavage sites (Exit module, Fig. 1) was designated the exit plasmid. The pEM106 exit plasmid was constructed and co-transformed with pEC101 elimination plasmid into *E. coli* NEB10β (Fig. 3b). At 12 h post-induction, the two plasmids were successfully and simultaneously eliminated from 75% of the cells (Fig. 3c), while the pEC101 plasmid was completely eliminated (Fig. 3d). The two-plasmid elimination was also confirmed by PCR amplification (Additional file 1: Figure S3). The results suggest that the EXIT circuit has potential to eliminate more plasmids simultaneously.

### Construction of the easy-to-use CRISPR-Cas9 system

**Fig. 4:**
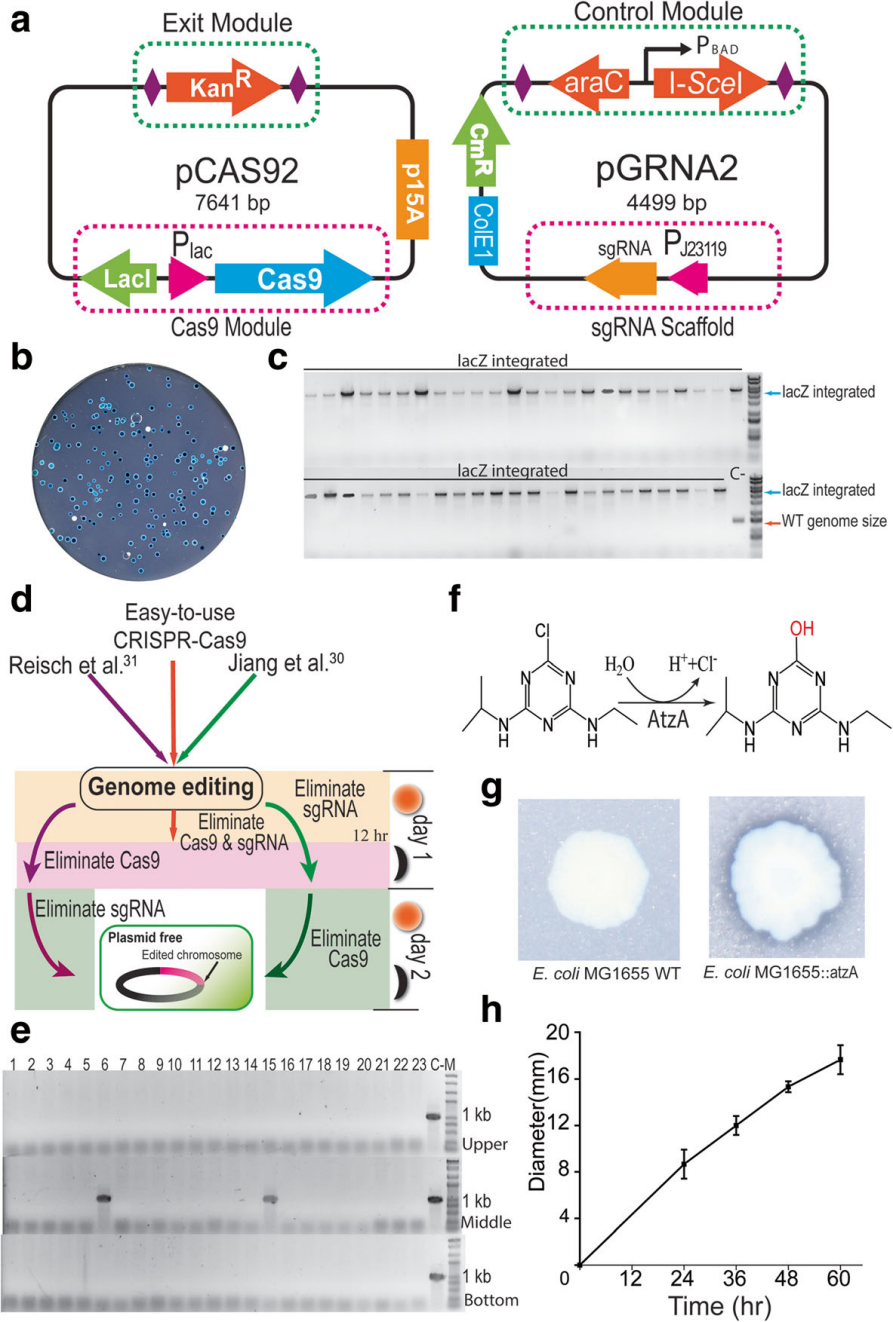
Construction and application of the easy-to-use CRISPR-Cas9 system. **a** Plasmid maps for the new easy-to-use CRISPR-Cas9 system. **b** Visual demonstration of efficient LacZ cassette integration via blue-white colony selection. **c** PCR verification of lacZ integration using a forward primer upstream of the left arm and a reverse primer downstream of the right arm of the chromosome. C-, with parent *E. coli* cells as the template. **d** Comparison of the easy-to-use CRISPR-Cas9 system versus previously developed CRISPR-Cas9 systems. The easy-to-use CRISPR-Cas9 system can eliminate plasmids in one single step instead of stepwise elimination. **e** PCR verification of plasmid elimination for pCAS92 (Upper), pGRNA2 (Middle) and pKD46 (Bottom). **f** AtzA catalyzes the hydrolytic dechlorination of atrazine to 2-hydroxyatrazine. **g** Atrazine degradation is indicated by the clear ring on an agar plate supplemented with atrazine. **h** The diameter of this degradation ring increased with extended incubation time

CRISPR-Cas9 systems were developed for genome editing in *E. coli* [8, 30–32], and the Cas9- and sgRNA-encoding plasmids persist in cells [8], or have to be eliminated sequentially [30–32] after genome editing. To solve this problem, we constructed a new and easy-to-use CRISPR-Cas9 system by patching the EXIT circuit, thus allowing one-step elimination of the two plasmids (Fig. 4a). The Cas9 protein under control of an IPTG-inducible P_lac_ promoter, and the exit module of the EXIT circuit were assembled in a p15A-origin plasmid, namely pCAS92. The sgRNA (under control of promoter P_J23119_) and the control module of the EXIT circuit were assembled in a ColE1-origin plasmid, namely pGRNA2. When genome editing was performed, the λ-Red recombinase was utilized to improve the frequency of homologous recombination (HR) [30, 31]. Additional file 1: Figure S4 shows the detailed procedure used for the easy-to-use CRISPR-Cas9 system.

We demonstrated the usefulness of the easy-to-use CRISPR-Cas9 system by editing the *E. coli* genome for scarless integration of the lacZ expression cassette. A target-sequence (5′-CGTAATATACGGGGTCAATA-3′) located between the *ompW* and *yciE* loci in the *E. coli* genome was chosen to build the pGRNA2-1 sgRNA plasmid (Additional file 1: Figure S5A). *E. coli* NEB10β, which harbors pCAS92 and pKD46, was co-transformed with pGRNA2-1, and the linear lacZ cassette with homology arms was supplied as a PCR product. The rescued colonies increased in number as the donor concentrations increased (Additional file 1: Figure S5B). The lacZ cassette integrated into the *E. coli* chromosome with 99.7% efficiency and formed blue colonies on 5-bromo-4-chloro-3-indolyl-β-D-galactopyranoside (X-gal) plates (Fig. 4b), a result confirmed by PCR amplification (Fig. 4c) and DNA sequencing (Additional file 1: Figure S6).

The advantage of the CRISPR-Cas9 system lies in its easy-to-use manner (Fig. 4d). One-step plasmid elimination is achievable unlike the stepwise elimination method of previously developed CRISPR-Cas9 systems [30–32]. The plasmids, pCAS92 and pGRNA2-1 were eliminated by induction with L-arabinose, and pKD46 was simultaneously eliminated by cultivation at 37 °C for 12 h [49]. Thus, all three plasmids were eliminated at one-step. Their elimination was achieved with high efficiency and was confirmed by PCR analysis. The plasmid pCAS92 (Fig. 4e, panel upper) and pKD46 (Fig. 4e, panel bottom) were eliminated from all 23 of the recombinant *E. coli* clones that were tested, while pGRNA2-1 was eliminated from 21 clones (Fig. 4e, middle panel).

We further utilized the easy-to-use CRISPR-Cas9 system to construct a scarless, plasmid-free atrazine degrading strain. Atrazine chlorohydrolase (AtzA) was able to catalyze the hydrolytic dechlorination of atrazine (Fig. 4f), yielding 2-hydroxyatrazine, which is non-herbicidal and non-phytotoxic [50]. AtzA driven by a strong promoter, was rapidly integrated into the wild-type *E. coli* MG1655 chromosome and the integration was confirmed (Additional file 1: Figure S7). Our results showed that the integrated AtzA was functionally expressed from the edited chromosome, and the recombinant *E. coli* acquired the ability to degrade atrazine. A clear ring resulting from the hydrolysis of atrazine was apparent on the agar plate supplemented with atrazine (Fig. 4g), and the diameter of this hydrolyzing ring increased as the incubation time was extended (Fig. 4h).

## Discussion

The CRISPR-Cas9 system has been widely used in genome-scale editing for microorganisms [8, 28–31, 49]. Upon completion of genome editing, removal of the CRISPR-Cas9 encoding plasmids is often essential for the next round of genome editing or to reuse the encoding plasmids with the same replication origins. Stepwise methods to eliminate CRISPR-Cas9-encoding plasmids have been reported [30–32]. With our newly established easy-to-use CRISPR-Cas9 system, the encoding plasmids were eliminated in a single step in 3–12 h. By comparison, the methods of Jiang et al. [30], Li et al. [32] and Reisch et al. [31] (Fig. 4d) reported that eliminating two encoding plasmids took at least 2 days and much more laboratory work was needed. Ronda et al. also exploited CRISPR-Cas9 cleavage ability to efficiently eliminate the gRNA plasmid, but did not demonstrated the Cas9 plasmid elimination [33]. Our easy-to-use CRISPR-Cas9 system significantly reduces the experimental workload and time requirements. Plasmid elimination using the current version of the easy-to-use CRISPR-Cas9 system is controlled by the same L-arabinose signal as in the original system, therefore the pCAS92 plasmid needs to be re-introduced into the cells when performing iterative genome editing. Thus, a modified version of the easy-to-use CRISPR-Cas9 system is needed in the future, where the EXIT circuits controlled by different signals would be patched to achieve sequential or simultaneous plasmid elimination as required. The λ-Red recombinase system has been demonstrated its ability to improve genome editing efficiency [30, 31]. In our present work, the λ-Red system was carried on a separate plasmid (pKD46) and worked together with the pCAS92 plasmid to enhance HR efficiency. Such λ-Red system is possibly assembled into the pCAS92 plasmid to build an all-in-one plasmid thus facilitate plasmid transformation and genetic manipulation.

In addition to the easy-to-use CRIPR-Cas9 system developed in this study, the newly designed EXIT circuit may have wider applications for plasmid elimination during genetic manipulation in various microorganisms. Physical and chemical methods [13, 14] are tedious to use and can damage chromosomal DNA molecules, while the methods that involve using counter-selection markers [15] or temperature-sensitive origins [16–18] usually involve construction of a host mutant [15] or construction of a replication origin mutant library with massive selection [51], all of which are labor-intensive and time-consuming. In contrast, the rationally designed EXIT circuit uses mild conditions and requires little labor. The EXIT circuit can also be used with other genome manipulation tools or strategies. We propose a new road map for developing next-generation tools and strategies for genome engineering involving the use of the EXIT circuit (Additional file 1: Figure S8). By patching the EXIT circuit, the plasmids in routine use can be modified as an easy-to-use plasmid kit. Through use of this kit, the plasmid-based genome engineering tools can be easily eliminated from hosts after genome manipulation is complete.

## Conclusion

In this study, we used the EXIT circuit as a proof of concept tool for plasmid elimination following genome editing. The EXIT circuit that is comprised of a control module and an exit module exploits the I-*Sce*I homing endonuclease and its cognate recognition site. Our data indicates that the EXIT circuit can be used for efficient and rapid elimination of one or more plasmids with diverse replication origins. The easy-to-use CRISPR-Cas9 system has been developed to allow for rapid, one-step elimination of Cas9- and the sgRNA-encoding plasmids. Although our work was carried out with *E. coli*, we consider that modified versions of the current EXIT circuit and the easy-to-use CRISPR-Cas9 system are potentially applicable to other microorganisms to bring about rapid plasmid elimination after genome editing.

## Methods

### Strains, plasmids, and cultivation conditions

The bacterial strains and plasmids used in this study are listed in Table 1. *E. coli* NEB10β was used for general plasmid construction and the validation experiments. WT *E. coli* MG1655 was used to construct the atrazine degradation strain. All *E. coli* strains were cultivated in liquid Luria-Bertani (LB) medium at 37 °C, or at 30 °C when pKD46 was present. When necessary, appropriate antibiotics were added to the cultures at the following concentrations (μg/mL): ampicillin (100), kanamycin (50), chloramphenicol (20), and erythromycin (200). L-arabinose was added to the medium as an inducer at the concentrations indicated. X-gal was plated onto LB agar for blue-white colony selection.

**Table 1:** Bacterial strains and plasmids used in this study

Strain or plasmid	Relevant characteristic(s)	References
Strains		
*Escherichia coli* NEB10β	Δ*(ara-leu) 7697 araD139 fhuA* Δ*lacX74 galK16 galE15 e14-* ϕ*80*d*lacZ*Δ*M15 recA1 relA1 endA1 nupG rpsL* (Str^R^) *rph spoT1* Δ*(mrr-hsdRMS-mcrBC)*	NEB
*E. coli* MG1655	K-12 F^−^ λ^−^ *ilvG* ^−^ *rfb-50 rph-1*	Lab stock
Plasmids		
pACYC184	p15A, Cm^R^	[42]
pCP202	Broad host vector pBBR1-MCS2 derivative, TET, P_CN_-GFP*mut3b*-T1T2	Lab stock
pKD46	Exo, bet, gam, rep and arabinose operon	[55]
pEC100	p15A, Cm^R^, TET-araC-P_BAD_-I-*Sce*I-T1T2	This study
pEC001	p15A, Cm^R^, araC-P_BAD_-I-*Sce*I-GFP*mut3b* fusion	This study
pEC101	p15A, Cm^R^, the control module composed of TET-araC-P_BAD_-I-*Sce*I-T1T2, bordered by two I-SceI cleavage sites	This study
pP103	p15A, Cm^R^, P_J23119_-GFPmut3b	Lab stock
pEC201	p15A, Cm^R^, the control module, P_J23119_-GFPmut3b	This study
pET-19(b)	pBR322, Amp^R^	Lab stock
pEC102	pBR322, Amp^R^, the control module	This study
pIC202	colE1, Cm^R^	Lab stock
pEC103	colE1, Cm^R^, the control module	This study
pUC-EM	pMB1, Em^R^	Lab stock
pEC104	pMB1, Em^R^, the control module	This study
pbeloBac11	repE, Cm^R^, sopA, sopB, sopC, cos	Lab stock
pEC105	pBeloBAC11 derivative, Cm^R^, the control module	This study
pEM106	pBR322, the exit module of Amp^R^	This study
pCAS9	Cas9	[8]
pICK1	p15A, Kan^R^, lacI-P_lac_-GFP	Lab stock
pICK2	p15A, lacI-P_lac_-GFP, the exit module of Kan^R^	This study
pCAS92	p15A, lacI-P_lac_-Cas9, the exit module of Kan^R^	This study
pGRNA2	colE, Cm^R^, sgRNA scaffold, the control module	This study
pGRNA2-1	pGRNA2 derivative, sgRNA with the N20 sequence targeting region between *ompW* and *yciE*	This study
pCM1001	incP broad host vector, P_TEF1_-lacZ-T1T2	This study
PCT100	Broad host vector pBBR1-MCS2 derivative, P_CN23_-atzA-T1T2	This study

### Genetic manipulation and transformation

Plasmid and chromosomal DNAs were isolated using the E.Z.N.A. Plasmid Mini Kit and the E.Z.N.A. bacterial DNA kit (OMEGA, Beijing, China). DNA fragments for plasmid assembly were purified using the E.Z.N.A. Gel Extraction Kit (OMEGA). PCR enzymes, Gibson reaction enzymes and restriction enzymes were purchased from New England BioLabs (MA, US). Q5 High-Fidelity DNA Polymerase was used for amplifying fragments for DNA assembly. PCR verification was performed with OneTaq 2X Master Mix. DNA sequencing was undertaken by Genewiz (Beijing, China).

*E. coli* electro-competent cells were prepared according to the method reported earlier [52]. Electroporation was performed in a pre-chilled 2 mm gap electroporation cuvette (Bio-Rad) at 2.5 kV with a Bio-Rad MicroPulser. LB (1 mL) was added to the shocked cells, which were allowed to recover for 1 h before plating them onto LB agar with the appropriate antibiotics.

### Plasmid construction

The Gibson assembly method [53] was used for plasmid construction. The resultant plasmids were confirmed by restriction enzyme digestion and sequencing. The pEC100 plasmid was constructed by amplifying terminator TET and terminator T1 T2 from plasmid pCP202, amplifying the araC-P_BAD_ cassette from plasmid pKD46, amplifying the I-*Sce*I open reading frame (ORF) from the synthesized template (Genewiz, Beijing), and cloning into pACYC184. The pEC001 I-*Sce*I-GFP*mut3b* fusion plasmid was constructed by inserting GFP*mut3b* into pEC100 downstream of I-*Sce*I. The control module of the EXIT circuit was constructed by inserting two I-SceI cleavage sites into both ends of the TET-araC-P_BAD_-I-*Sce*I-T1 T2 cassette of pEC100 to generate plasmid pEC101. The GFP*mut3b* pEC201 indicator plasmid was constructed by amplifying the P_J23119_-driven GFP*mut3b* reporter gene from plasmid pP103 and inserting it into plasmid pEC101. The control module was assembled in pbeloBac11 to generate plasmid pEC105, in pET-19(b) (pBR322 origin) to generate plasmid pEC102, in plasmid pIC202 (ColE1 origin) to generate plasmid pEC103, and in plasmid pUC-EM (pMB1) to generate plasmid pEC104. The exit module pEM106-encoding plasmid was constructed by introducing two I-*Sce*I cleavage sites at both ends of the original ampicillin marker in plasmid pET-19(b).

To construct the easy-to-use CRISPR-Cas9 system, two I-SceI cleavage sites were introduced to both ends of the kanamycin cassette of plasmid pICK1 to generate plasmid pICK2. The Cas9 protein-encoding gene was then amplified from pCas9 to replace the GFP ORF of pICK2, resulting in plasmid pCAS92. The sgRNA scaffold, together with the strong P_J23119_ promoter, was synthetized by Genewiz (Beijing, China), after which it was PCR-amplified and cloned into plasmid pEC103 to generate plasmid pGRNA2. A DNA annealing procedure was used to obtain the N20 pairing of the targeted genome sequence. The 20 nucleotide sequence region (N20, Additional file 1: Figure S5) between *ompW* and *yciE* was introduced using the primers that amplified the pGRNA2 scaffold to generate plasmid pGRNA2-1.

### Validating the control module at population and single-cell levels

An overnight culture of *E. coli* NEB10β/pEC001 or *E. coli* NEB10β/pEC201 was inoculated into LB broth with different concentrations of L-arabinose, and then transferred to separate wells in a 96-well flat clear-bottom plate (Corning Costar, cat. # 3603). The plate was incubated at 37 °C overnight on a microplate reader (BioTek, SynergyH4) and measurements were taken. GFP fluorescence was continuously read (excitation wavelength of 488 nm; emission wavelength of 520 nm), and cellular growth was detected at 600 nm (optical density, OD_600_).

An overnight culture of *E. coli* NEB10β/pEC201 was inoculated to LB broth with 100 mM L-arabinose, and samples were taken every 6 h. One mL samples were spun down and the pellets were re-suspended in 1 mL of PBS and then stored on ice until analysis. The GFP distribution in the cell population was analyzed by flow cytometry using a BD FACS Calibur cytometer. For each sample, 30,000 events were analyzed. Data processing was performed using Flowjo7.5.

### Plasmid elimination procedure and elimination efficiency determination

*E. coli* for the plasmids patching the EXIT circuit were induced with L-arabinose and then plated onto antibiotic-free plates. The colonies grown up were picked and streaked onto plates containing the appropriate antibiotics for growth tests. Antibiotic-sensitive colonies were considered to have had their plasmid(s) eliminated. The results were confirmed by PCR verification with plasmid-specific primer pairs.

To determine the elimination efficiency, 100 colonies formed on antibiotic-free plates were picked for streaking onto LB broth containing appropriate antibiotics. Colonies with sensitivity to the appropriate antibiotics were counted as those with successfully eliminated plasmids (number of sensitive colonies per total number of colonies tested). The experiments were performed in triplicate.

### Genome editing procedure

An overnight culture of *E. coli* harboring both pCAS92 and pKD46 was added to fresh LB medium containing ampicillin and kanamycin for culturing at 30 °C with 150 rpm. When the OD_600_ reached 0.25–0.30, 1 mM IPTG was added to induce Cas9 expression, and 10 mM L-arabinose was added to induce λ-Red recombinase expression. When the OD_600_ reached 0.55–0.60, the cells were harvested to prepare electro-competent cells. The cells (50 μL) were mixed with 100 ng of the sgRNA plasmid and different amounts (as indicated) of the linear dsDNA donor supplied as a PCR product, and electroporation was performed. After 2 h recovery at 30 °C, the culture was plated onto LB agar containing kanamycin and chloramphenicol. The colonies formed were confirmed as integration positive by colony PCR with a forward primer upstream of the left homology arm and a reverse primer downstream of the right homology arm. *E. coli* parent strains were used as negative controls. PCR products of the correct sizes were subjected to DNA sequencing for verification.

Donor DNA was prepared as follows: The P_TEF1_-lacZ-T1T2 cassette was PCR-amplified from plasmid pCM1001, and the P_CN23_-atzA-T1T2 cassette was PCR-amplified from plasmid pCT100. Both were assembled with 600-bp upstream and downstream homology arms to assist the integration site amplified from boiled *E. coli* cells through splicing by overlapping extension PCR [54].

After genome editing, the plasmids were eliminated in one step by growing the host cells at 37 °C and inducing them with saturated L-arabinose. After 12 h cultivation, the cells were diluted and plated onto antibiotic-free plates. The colonies formed were confirmed to be plasmid eliminated by PCR verification with the following three sets of primer pairs: CON.PCAS92 F/R, CON.PGRNA2 F/R, and CON.PKD46 F/R.

### Integration efficiency determination and rescue ability calculations

The donor DNA for the P_TEF1_-lacZ-T1T2 cassette was mixed with pGRNA2-1 and co-transformed into *E. coli* NEB10β cells harboring pCAS92 and pKD46. After recovery, the cells were plated onto X-gal plates for blue-white colony selection. Blue colonies were considered to be successfully integrated ones (number of blue colonies/total number of colonies). The rescue ability of different amounts of donor DNA was calculated by counting the number of colony forming units (CFUs) in the experimental plates (i.e., cotransformation of the linear lacZ template and genome targeting gRNA) with a control plate (transformation of the genome targeting gRNA only).

### Atrazine degradation strain construction and atrazine degradation plate assays

The donor DNA for the P_CN23_-atzA-T1T2 cassette was mixed with sgRNA plasmid pGRNA2-1 and then cotransformed into *E. coli* MG1655, which harbored pCAS92 and pKD46. The resultant colonies were confirmed by PCR and the atzA-integrated clone had eliminated its plasmids. Atrazine was spread onto solid LB plates at 100 μg per mL to produce an opaque LB agar plate. Atrazine degradation was indicated by a clear ring around the atrazine-degraded colonies.

## Additional file

Additional file 1: Figure S1. Single-cell level evaluation of the control module of EXIT circuit at 12-h and 18-h point. **Figure S2.** PCR verification for elimination of plasmid pEC101 (A), pEC102 (B), pEC103 (C), pEC104 (D), and pEC105 (E). DDW: with sterilized water as template; C-:with parent E. coli harboring the corresponding plasmid as template. **Figure S3.** PCR verification of two plasmid elimination. Lane 1, amplification with primer pair CON.PEC101 F/R; Lane 2, PCR amplification with primer pair CON.PEM106 F/R. DDW: template with sterilized water as control; C-:with parent E. coli harboring plasmid pEC101 and pEM106 as template. **Figure S4.** Detailed diagram of genome editing procedure with the easy-to-use CRISPR-Cas9 system. **Figure S5**. Genome integration mediated by the easy-to-use CRISPR-Cas9 system. (A) The design of single-guide RNA for targeted site integration. (B) The Rescue ability relative to donor concentrations. 3 samples were determined and the standard errors are indicated. **Figure S6.** DNA sequencing of the upstream (A) and downstream (B) for confirmation of lacZ cassette integration. **Figure S7.** DNA sequencing for confirmation of atzA cassette integration. **Figure S8.** New roadmap proposed for developing next-generation genetic editing tools and strategies. **Table S1.** Primers used in this study. (DOC 9702 kb)

## Abbreviations

Amp^R^: Ampicillin resistance gene
CFUs: Colony forming units
Cm^R^: Chloramphenicol resistance gene
CRISPR: Clustered regularly interspaced short palindromic repeats
DSBs: Double-strand DNA breaks
*E. coli*: *Escherichia coli*
Em^R^: Erythromycin resistance gene
FACS: Fluorescence activate cell sorting
HEG: Homing endonuclease gene
HR: Homologous recombination
IPTG: Isopropyl β-D-1-thiogalactopyranoside
Kan^R^: Kanamycin resistance gene
N20: 20-nt region complementary to the targeting region
ORF: Open reading frame
PAM: Protospacer adjacent motif
PBS: Phosphate buffered saline
PCR: Polymerase chain reaction
X-gal: 5-Bromo-4-chloro-3-indolyl-β-D-galacto-pyranoside
