# Supplementary material for: Construction of an easy-to-use CRISPR-Cas9 system by patching a newly designed EXIT circuit
Source: J Biol Eng. 2017 Sep 4;11:32. doi: 10.1186/s13036-017-0072-5 (PMC5582390; doi:10.1186/s13036-017-0072-5)
Supplement: Additional file 1: Figure S1. — Single-cell level evaluation of the control module of EXIT circuit at 12-h and 18-h point. Figure S2. PCR verification for elimination of plasmid pEC101 (A), pEC102 (B), pEC103 (C), pEC104 (D), and pEC105 (E). DDW: with sterilized water as template; C-:with parent E. coli harboring the corresponding plasmid as template. Figure S3. PCR verification of two plasmid elimination. Lane 1, amplification with primer pair CON.PEC101 F/R; Lane 2, PCR amplification with primer pair CON.PEM106 F/R. DDW: template with sterilized water as control; C-:with parent E. coli harboring plasmid pEC101 and pEM106 as template. Figure S4. Detailed diagram of genome editing procedure with the easy-to-use CRISPR-Cas9 system. Figure S5. Genome integration mediated by the easy-to-use CRISPR-Cas9 system. (A) The design of single-guide RNA for targeted site integration. (B) The Rescue ability relative to donor concentrations. 3 samples were determined and the standard errors are indicated. Figure S6. DNA sequencing of the upstream (A) and downstream (B) for confirmation of lacZ cassette integration. Figure S7. DNA sequencing for confirmation of atzA cassette integration. Figure S8. New roadmap proposed for developing next-generation genetic editing tools and strategies. Table S1. Primers used in this study. (DOC 9702 kb) [file 13036_2017_72_MOESM1_ESM.doc]

Construction of an easy-to-use CRISPR-Cas9 system by patching a newly designed EXIT circuit

Qiang Tang^1,2^ Chun-bo Lou^3^* Shuang-Jiang Liu^1^*

^1^State Key Laboratory of Microbial Resources and Environmental Microbiology Research Center, Chinese Academy of Sciences, Beijing, 100101, China;

^2^University of Chinese Academy of Sciences, Beijing 100049, China;

^3^CAS Key Laboratory for Microbial Physiology and Metabolic Engineering, Chinese Academy of Sciences, Beijing, 100101, China

*Corresponding authors: Shuang-Jiang Liu and Chun-bo Lou

Institute of Microbiology

Chinese Academy of Sciences

Beichen Xilu 1, Chaoyang District

Beijing 100101, China

Tel: +86-10-64807423

Fax: +86-10-64807421

Email: SL (liusj@im.ac.cn);

CL (louchunbo@im.ac.cn);
QT (tomimcas@gmail.com)

1. Additional file figures


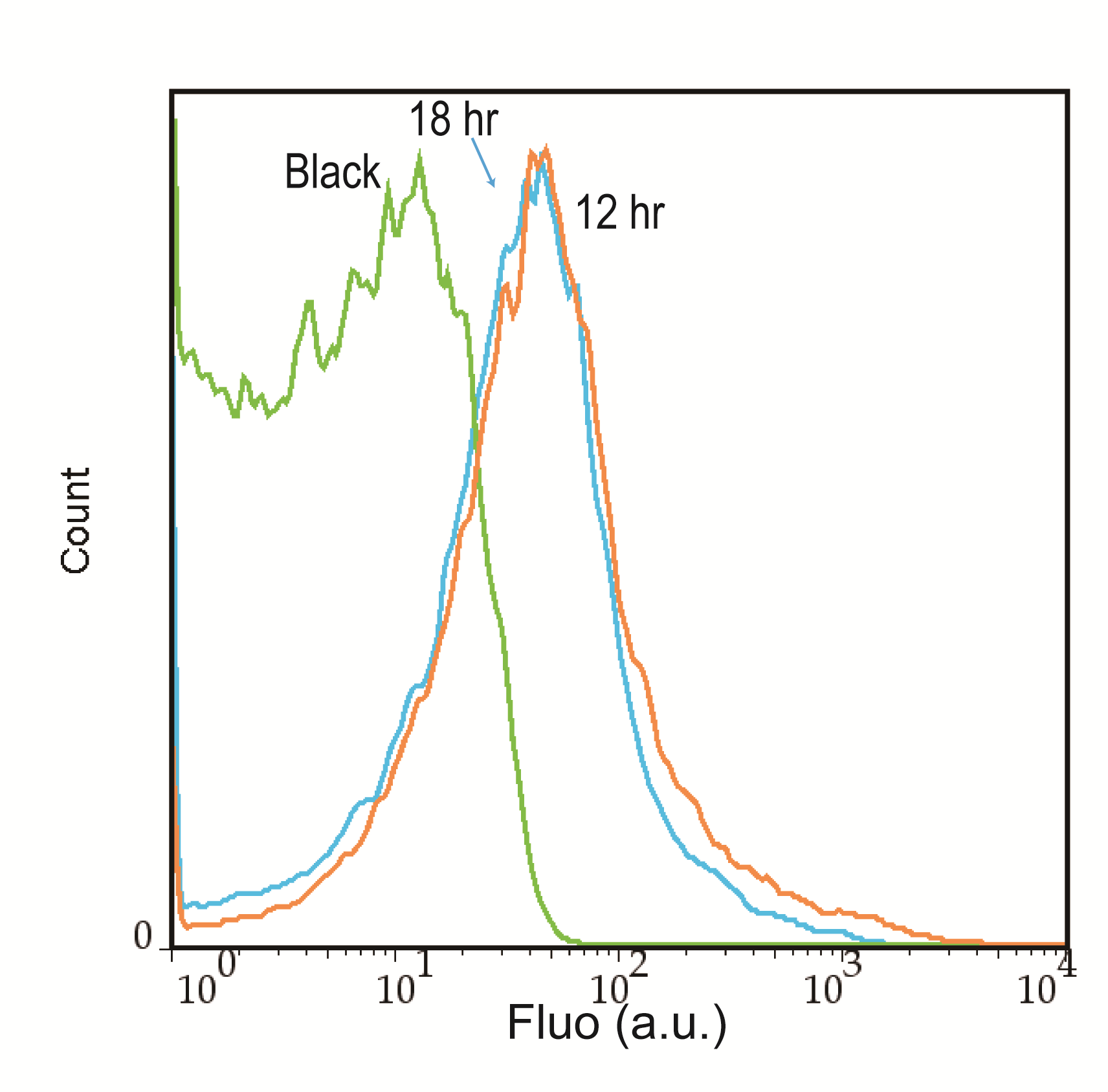


Fig. S1. Single-cell level evaluation of the control module of EXIT circuit at 12-hr and 18-hr point


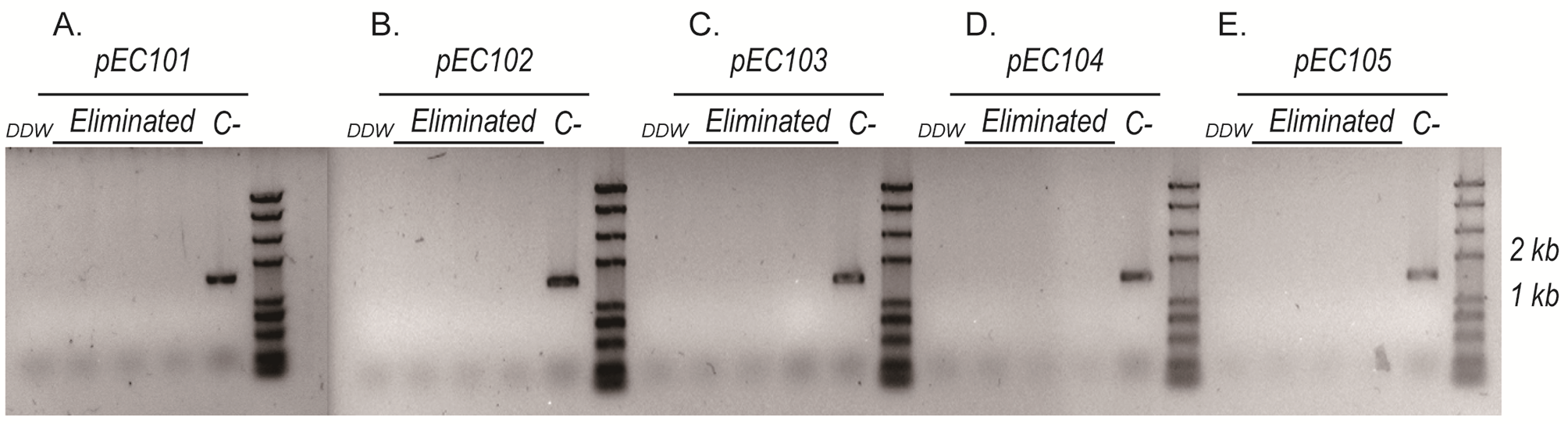


Fig. S2. PCR verification of plasmid elimination. (A) Elimination of plasmid pEC101. PCR with primer pair CON.PEC101 F/R. (B) Elimination of plasmid pEC102. PCR with primer pair CON.PEC102 F/R. (C) Elimination of plasmid pEC103. PCR with primer pair CON.PEC103 F/R. (D) Elimination of plasmid pEC104. PCR with primer pair CON.PEC104 F/R. (E) Elimination of plasmid pEC105. PCR with primer pair CON.PEC101 F/R. DDW: with sterilized water as template; C-：with parent *E. coli* harboring the corresponding plasmid as template.


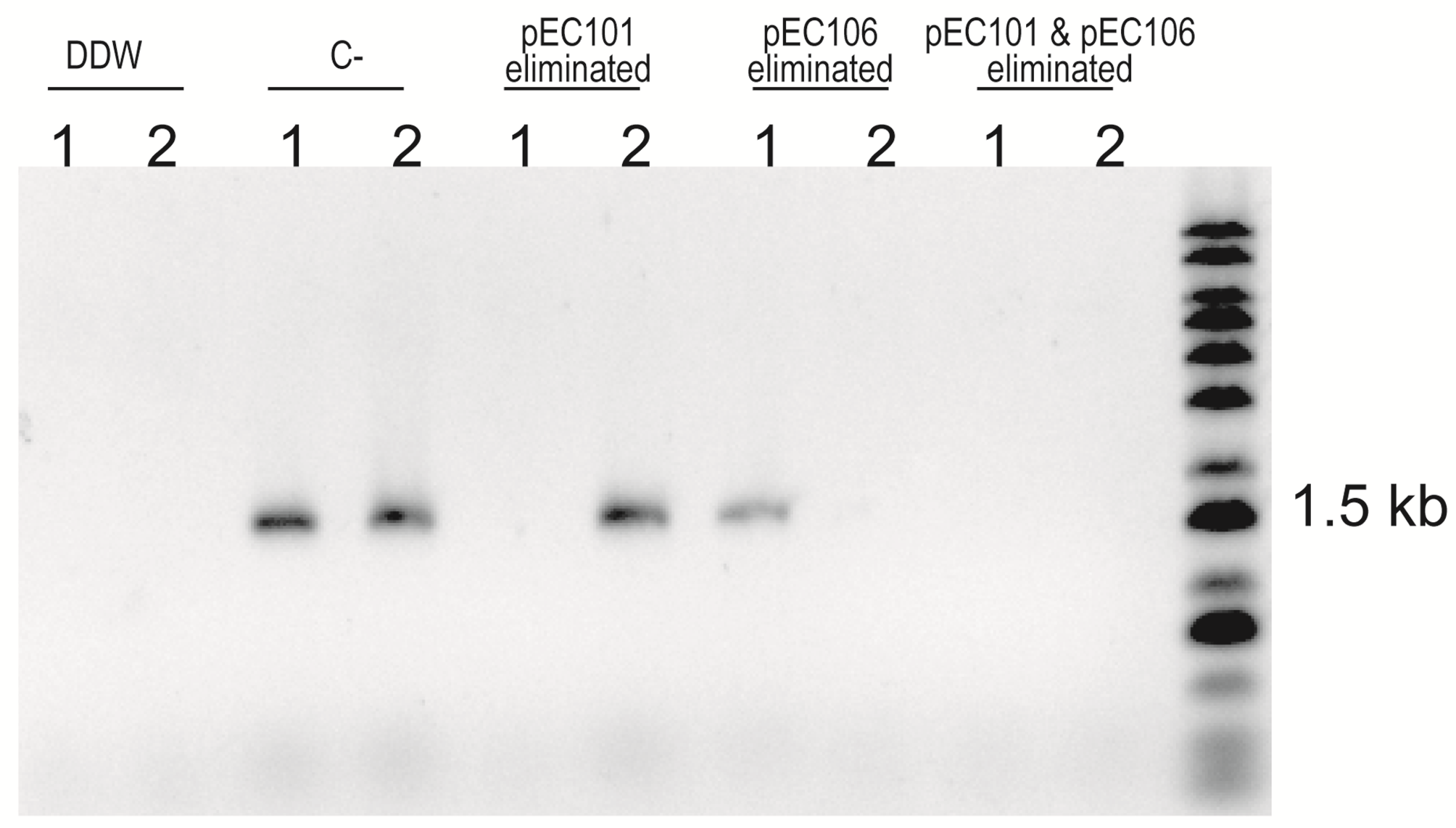


Fig. S3. PCR verification of two plasmid elimination. 1, PCR amplification with primer pair CON.PEC101 F/R; 2, PCR amplification with primer pair CON.PEM106 F/R. DDW: template with sterilized water as control; C-：with parent *E. coli* harboring plasmid pEC101 and pEM106 as template; pEC101 eliminated: with *E. coli* eliminated pEC101 but pEM106 remained as template; pEM106 eliminated: with *E. coli* eliminated pEM106 but pEC101 remained as template; pEC101 & pEM106 eliminated: with both pEC101 and pEM106 eliminated as template.


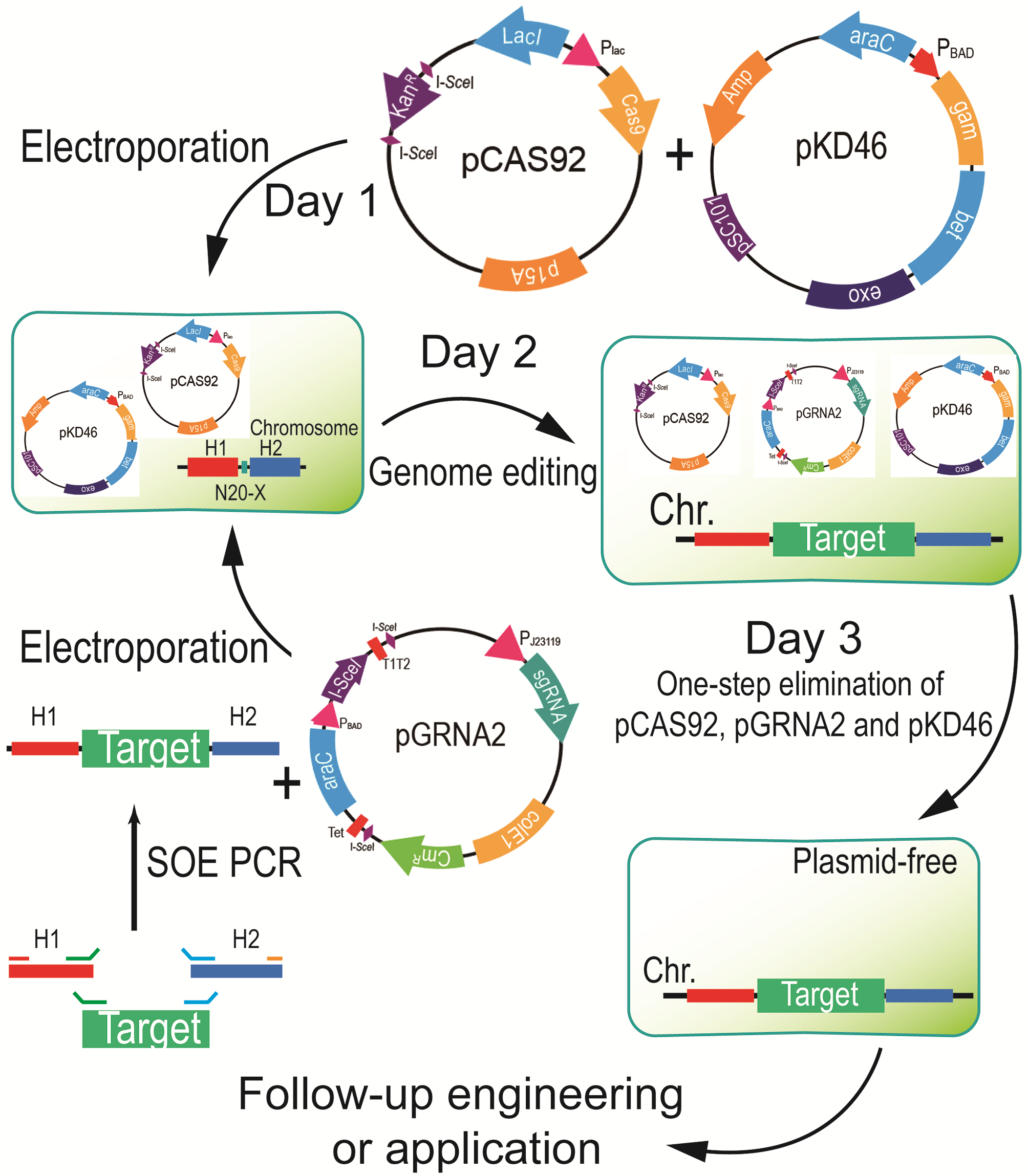


Fig. S4. Detailed diagram of genome editing procedure with the easy-to-use CRISPR-Cas9 system


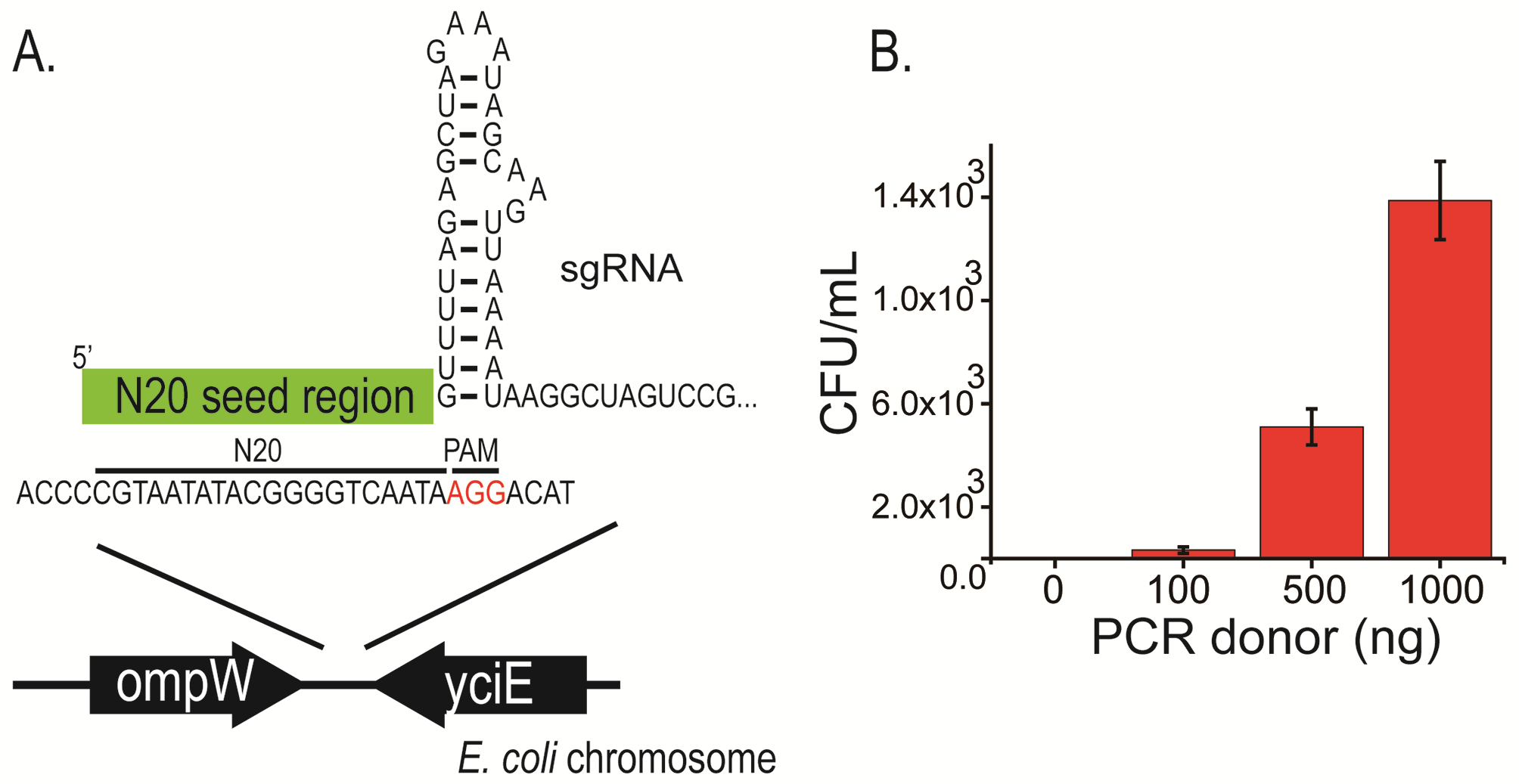


Fig. S5. Genome integration mediated by the easy-to-use CRISPR-Cas9 system. (A) The design of single-guide RNA for targeted site integration. (B) The Rescue ability relative to donor concentrations. 100 ng sgRNA and different amounts of PCR donors (0, 100, 500 and 1000 ng) were co-transformed and formed CFUs were counted. 3 samples were determined and the standard errors are indicated.


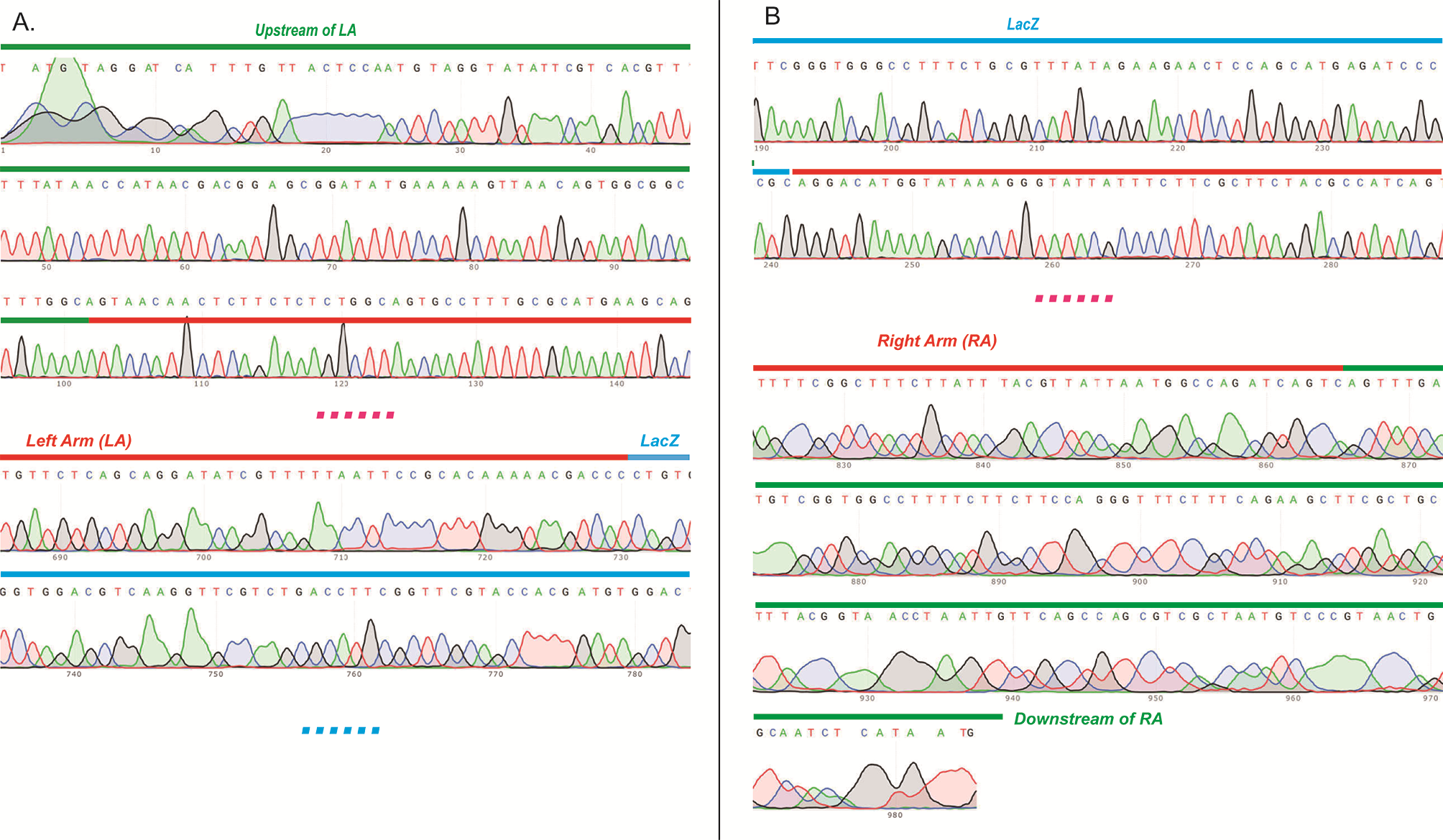


Fig. S6. DNA sequencing of the upstream (A) and downstream (B) for confirmation of lacZ cassette integration


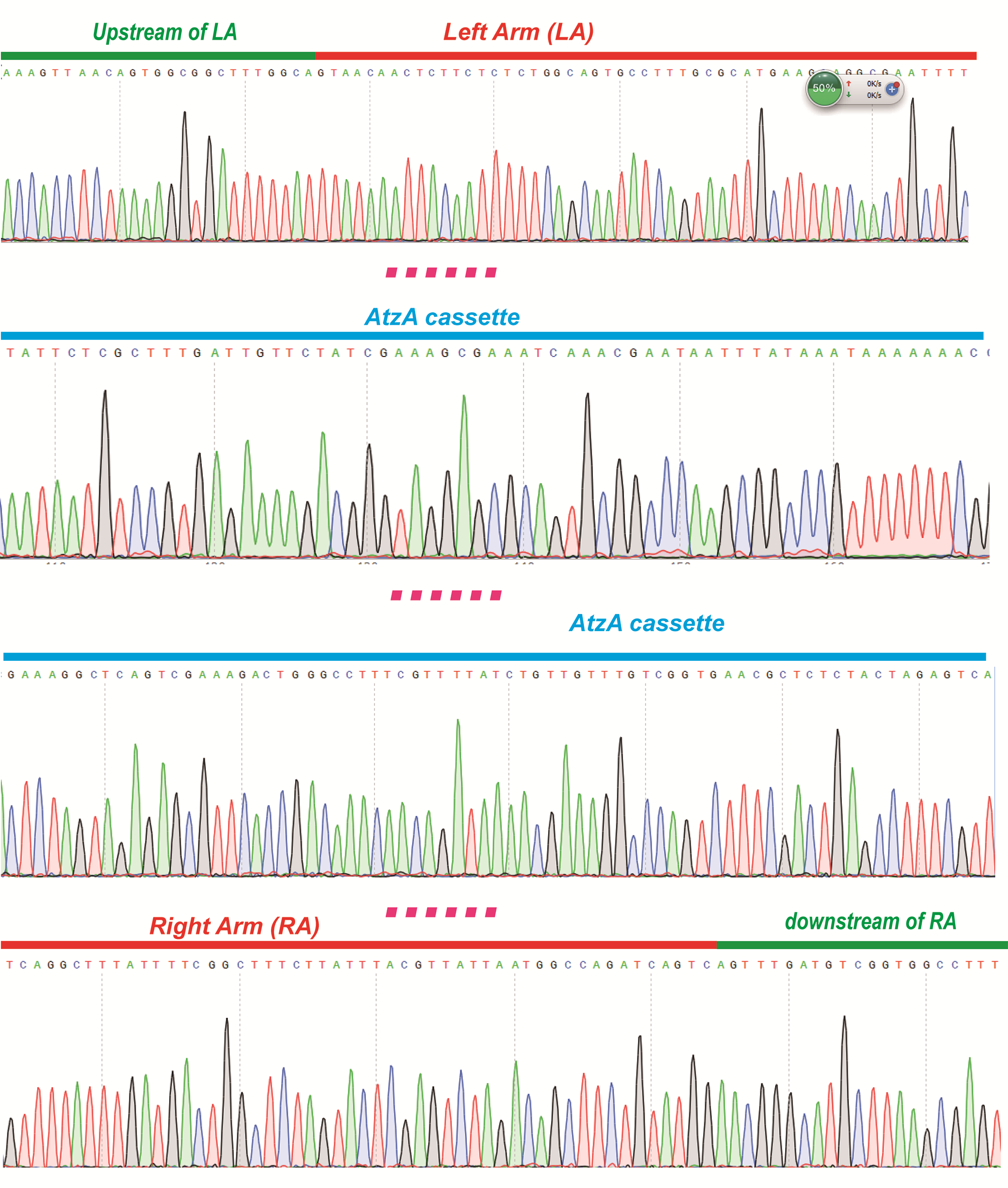


Fig. S7. DNA sequencing for confirmation of atzA cassette integration


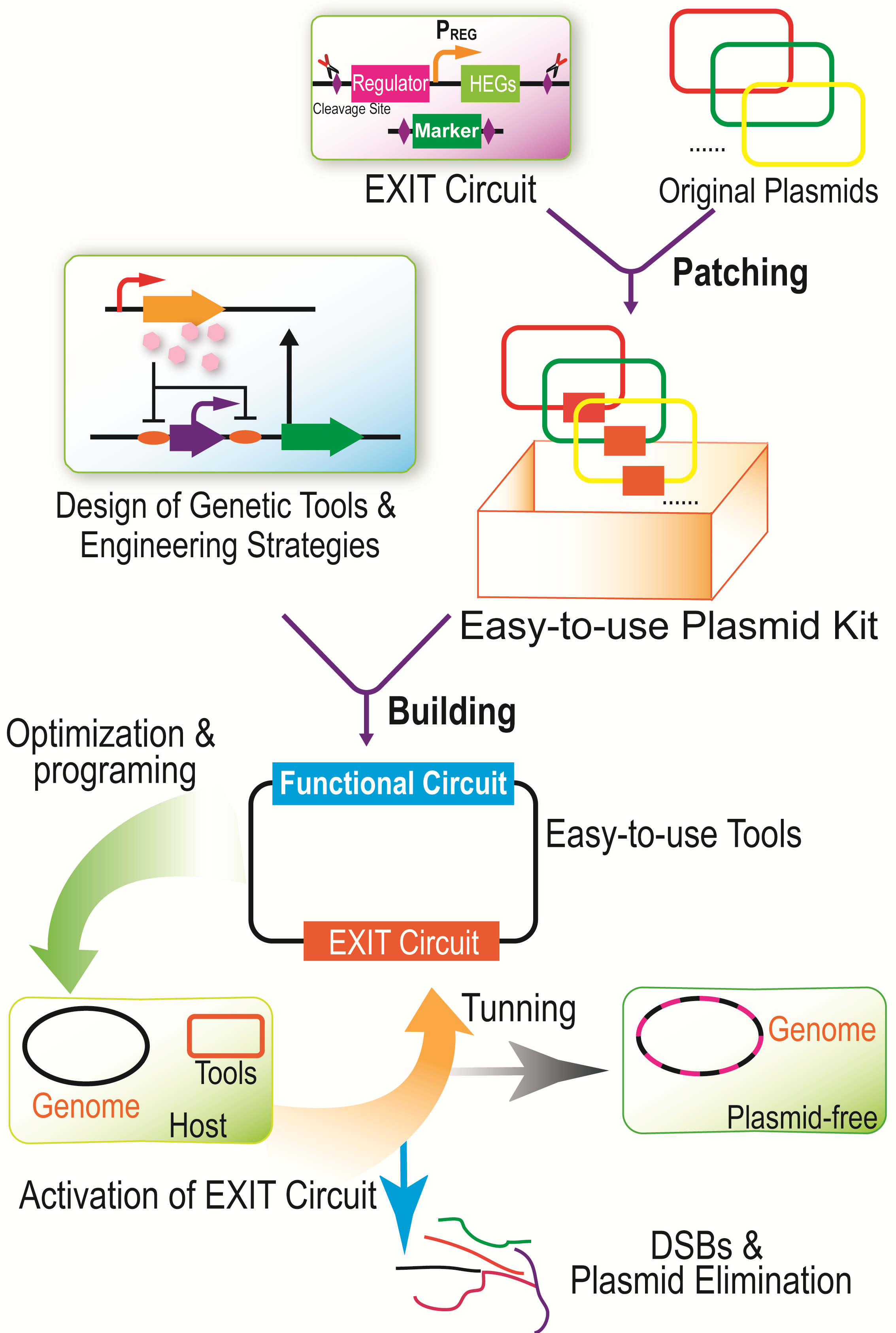


Fig. S8. New roadmap proposed for developing next-generation genetic editing tools and strategies. The original plasmids are modified as an easy-to-use plasmid kit by pathching the EXIT circuit. Newly designed genetic tools or strategies are built on the plasmid kit. The resultant tool plasmid allows for easy elimination upon the completion of the genetic editing

1. Additional file table

Table S1. Primers used in this study

| Primers | Sequences (5’-3’) | Description |
| --- | --- | --- |
| T1T2-P15A F | CGGGTGGGCCTTTCTGCGTTTATACCGACGACCGGGTCGAATTTGC | Construct pEC100 |
| TET-P15A R | CAGAATGTCCGAGACTAATTCATGATTGAGATCGTTTTGGTCTGCGCGTAATC |
| P15A-TET F | CGCGCAGACCAAAACGATCTCAATCATGAATTAGTCTCGGACATTCTG |
| PBAD-TET R | AATGATGTAGCCGTCAAGTTGTCATAAGAATTCCTCGAGTCTAGAGGAGCATGC |
| TET-PBAD F | ATGCTCCTCTAGACTCGAGGAATTCTTATGACAACTTGACGGCTACATCATTC |
| ISCEI-PBAD R | ACCTGGTTTTTTTGATGCATTTTTTATAACCTCCTTAGAGCTCG |
| PBAD-ISCEI F | CTAAGGAGGTTATAAAAAATGCATCAAAAAAACCAGGTAATG |
| TET-ISCEI R | GGCTCTAGTACTCGAGTCTAGATTATTTCAGGAAAGTTTCGGAGGAGATAG |
| ISCEI-T1T2 F | CCTCCGAAACTTTCCTGAAATAATCTAGACTCGAGTACTAGAGCCAGGC |
| P15A-T1T2 R | GCAAATTCGACCCGGTCGTCGGTATAAACGCAGAAAGGCCCACCCG |
| P15-CONT F | GAAGTGTAGGAGCCCTAGGGATAACAGGGTAATTCATGAATTAGTCTCGGACATTCTG | Amplify Control Module |
| P15-CONT R | GCCAGTAGAATCCGTATTACCCTGTTATCCCTATATAAACGCAGAAAGGCCCACCCG |
| CONT-P15 F | ACGGATTCTACTGGCCCGACGACCGGGTCGAATTTGC | Construct pEC101 |
| CONT-P15 R | GGGCTCCTACACTTCTTGAGATCGTTTTGGTCTGCGCGTAATC |
| CONT-PBR32 F | ACGGATTCTACTGGCGCCTTCCCCATTATGATTCTTCTCGC | Construct pEC102 |
| CONT-PBR32 R | GGGCTCCTACACTTCTTCTTGAAGACGAAAGGGCCTCGTG |
| CONT-COLE F | ACGGATTCTACTGGCCTAGGGGATATATTCCGCTTCCTCG | Construct pEC103 |
| CONT-COLE R | GGGCTCCTACACTTCTCGGCACGTAAGAGGTTCCAAC |
| CONT-PMB F | ACGGATTCTACTGGCCGGTTTGCGTATTGGGCGCTC | Construct pEC104 |
| CONT-PMB R | GGGCTCCTACACTTCTCATTAATGCAGCTGGCACGAC |
| CONT-PBELO F | ACGGATTCTACTGGCCCAGGCGTTTAAGGGCACCAATAAC | Construct pEC105 |
| CONT-PBELO F | GGGCTCCTACACTTCGTCGACAGCGACACACTTGCATC |
| PBR322-EM F | ACTACTGACTGGGATAATCTTAGGGATAACAGGGTAATGACGAAAGGGCCTCGTGATACG | Construct pEM106 |
| PBR322-EM R | CGTGCTTTCAACAATAGGTAATTACCCTGTTATCCCTACGCTCAGTGGAACGAAAACTCACG |
| EM-PBR322 F | TACCTATTGTTGAAAGCACGTCAGACCCCGTAGAAAAGATC |
| EM-PBR322 R | AGATTATCCCAGTCAGTAGTTCGCGCGTTTCGGTGATG |
| PEC101-GFP F | GAATAAGTGATAATAAGCGGATGAATGGCATTGACAGCTAGCTCAGTCCTAGGTATAATG | Construct pEC201 |
| PEC101-GFP R | GTTGGAAACTCTACGATTTAGCCTGCCCGGTTATTA |
| GFP-PEC101 F | TAAATCGTAGAGTTTCCAACGAAATTCGAAAGCAAATTCG |
| GFP-PEC101 R | CATTATACCTAGGACTGAGCTAGCTGTCAATGCCATTCATCCGCTTATTATCACTTATTC |
| CON.PEC101 F | CTGGCTTGGAGGAGCGCAG | Confirm pEC101 elimination |
| CON.PEC101 R | ACCCAGGGATTGGCTGAGACG |
| CON.PEC102 F | CAGCTTCAAGGATCGCTCGCG | Confirm pEC102 elimination |
| CON.PEC102 R | GTCGGAACAGGAGAGCGCACG |
| CON.PEC103 F | GCGTTGCTGGCGTTTTTCCATAGGC | Confirm pEC103 elimination |
| CON.PEC103 R | TCGGCACGTAAGAGGTTCCAAC |
| CON.PEC104 F | CGCAGGAAAGAACATGTGAGC | Confirm pEC104 elimination |
| CON.PEC104 R | CCGATACCGTTTACGAAATTGGAAC |
| CON.PEC105 F | TACGCCCCGCCCTGCCACTC | Confirm pEC105 elimination |
| CON.PEC105 R | TGCTGACATAATCCGCTCCACTTC |
| CON.PEM106 F | TACGGGGTCTGACGCTCAGTGGAAC | Confirm pEM106 elimination |
| CON.PEM106 R | CAGGGTCGGAACAGGAGAGC |
| GRNA-PEC103 F | CTTCAGCATGTTCAATGATGTCGCTCACTGACTCGCTACAGTAAAGGC | Construct pGRNA2 |
| GRNA-PEC103 R | ACTGAGCTAGCTGTCAACGAGGAAGCGGAATATATCCCCTAG |
| PEC103-GRNA F | CTAGGGGATATATTCCGCTTCCTCGTTGACAGCTAGCTCAGTCCTAGGT |
| PEC103-GRNA R | GCCTTTACTGTAGCGAGTCAGTGAGCGACATCATTGAACATGCTGAAG |
| GRNA1-GPLAT F | CGTAATATACGGGGTCAATAGTTTTAGAGCTAGAAATAGCAAGTTAAAATAAGGC | Construct pGRNA-1 |
| GRNA1-GPLAT R | TATTGACCCCGTATATTACGGCTAGCATTATACCTAGGACTGAGCTAGC |
| PICK1-IKAN F | CGTGCTTTCAACAATAGGTAATTACCCTGTTATCCCTACTCTCGAACCCCAGAGTCCCG | Construct pICK2 |
| PICK1-IKAN R | AGATTATCCCAGTCAGTAGTTAGGGATAACAGGGTAATCGTCGGAATTGCCAGCTGGGGC |
| IKAN-PICK F | ACTACTGACTGGGATAATCTTCATTGCGTTGCGCTCACTGCCCGCTTTCCAGTC |
| IKAN-PICK R | TACCTATTGTTGAAAGCACGCGCTTGGACTCCTGTTGATAGATCCAG |
| PICK2-CAS9 F | TACCTAGAATTAAAGAGGAGAAATTAAGCATGGATAAGAAATACTCAATAGGCTTAGATATC | Construct pCAS92 |
| PICK2-CAS9 R | AGGTCAGCTAATTAAGCTTATCAGTCACCTCCTAGCTGACTC |
| CAS9-PICK2 F | GAGTCAGCTAGGAGGTGACTGATAAGCTTAATTAGCTGACCTACTAGTCGGCCG |
| CAS9-PICK2 R | TATCTAAGCCTATTGAGTATTTCTTATCCATGCTTAATTTCTCCTCTTTAATTCTAGGTA |
| CON.PCAS92 F | AGAACCTGCGTGCAATCCATC | Confirm pCAS92 elimination |
| CON.PCAS92 R | GCGTGGTGGTGTCGATGGTAG |
| CON.PGRNA2 F | ACCGTACCTGATCCCGCAGATG | Confirm pGRNA2 elimination |
| CON.PGRNA2 R | GCGGACAGGTATCCGGTAAGC |
| CON.PKD46 F | AGCAGCTCGCCCGTGAAGAG | Confirm pKD46 elimination |
| CON.PKD46 R | ATGCTGCCACCTTCTGCTCTGC |
| CON.UPINT F | GGCGACCGAGTTAATATTTGCGTAGCG | Confirm integration between *ompW* and *yciE* |
| CON.UPINT R | AAGTGCGTGATGCCGCACTG |
| LARM F | GTAACAACTCTTCTCTCTGGCAG | Construct donor PCR product |
| TET-LARM R | GCAGAATGTCCGAGACTAATTCATGAGGGTCGTTTTTGTGCGGAATTAAAAAC |
| LARM-TET F | TTTTTAATTCCGCACAAAAACGACCCTCATGAATTAGTCTCGGACATTCTGC |
| RARM-T1T2 R | GAAATAATACCCTTTATACCATGTCCTGCGGGGATCTCATGCTGGAG |
| T1T2-RARM F | CTCCAGCATGAGATCCCCGCAGGACATGGTATAAAGGGTATTATTTC |
| RARM R | GACTGATCTGGCCATTAATAACG |
